# Overcoming immunogenicity issues of HIV p24 antigen by the use of innovative nanostructured lipid carriers as delivery systems: evidences in mice and non-human primates

**DOI:** 10.1038/s41541-018-0086-0

**Published:** 2018-10-01

**Authors:** Emilie Bayon, Jessica Morlieras, Nathalie Dereuddre-Bosquet, Alexis Gonon, Leslie Gosse, Thomas Courant, Roger Le Grand, Patrice N. Marche, Fabrice P. Navarro

**Affiliations:** 1 Univ. Grenoble Alpes, F-38000 Grenoble, France; 2 Division for biology and healthcare technologies, CEA, LETI, MINATEC Campus, F-38054 Grenoble, France; 3 INSERM U1209, IAB, F-38700 La Tronche, France; 4 Center for Immunology of Viral Infections and Autoimmune Diseases, IDMIT department, IBFJ, CEA, Université Paris Sud 11, INSERM U1184, 92265 Fontenay-aux-Roses, France

## Abstract

HIV is one of the deadliest pandemics of modern times, having already caused 35 million deaths around the world. Despite the huge efforts spent to develop treatments, the virus cannot yet be eradicated and continues to infect new people. Spread of the virus remains uncontrolled, thus exposing the worldwide population to HIV danger, due to the lack of efficient vaccines. The latest clinical trials describe the challenges associated with developing an effective prophylactic HIV vaccine. These immunological obstacles will only be overcome by smart and innovative solutions applied to the design of vaccine formulations. Here, we describe the use of nanostructured lipid carriers (NLC) for the delivery of p24 protein as a model HIV antigen, with the aim of increasing its immunogenicity. We have designed vaccine formulations comprising NLC grafted with p24 antigen, together with cationic NLC optimized for the delivery of immunostimulant CpG. This tailored system significantly enhanced immune responses against p24, in terms of specific antibody production and T-cell activation in mice. More importantly, the capacity of NLC to induce specific immune responses against this troublesome HIV antigen was further supported by a 7-month study on non-human primates (NHP). This work paves the way toward the development of a future HIV vaccine, which will also require the use of envelope antigens.

## Optimizing HIV p24 vaccine responses

To date, HIV vaccines have resulted in poor or absent protection. A team led by Fabrice P. Navarro at the CEA LETI use the conserved HIV capsid protein p24 vectorized into cationic nanostructured lipid carriers (NLC-p24) along with NLC-delivered CpG. Owing to their small size, NLCs gain access to lymph nodes and deliver antigen directly to antigen presenting cells. Anti-p24 responses have been associated with effective HIV control, making them an attractive vaccine antigen, but they are poorly immunogenic. NLC-p24 shows a good safety profile while at the same time being able to elicit robust humoral and cellular immune responses in both mice and *Cynomolgus* macaques. NLC-mediated delivery of both p24 and CpG results in more effective immune stimulation than delivery of free antigen and adjuvant. These findings demonstrate the possibility of priming effective responses to a potent but otherwise poorly immunogenic HIV antigen.

## Introduction

Fighting the HIV pandemic is one of the major priorities for healthcare worldwide. According to UNAIDS statistics from 2016, HIV has already caused 35 million deaths around the world, with 76 million people having been infected since the beginning of the epidemic in the 1970s. For three decades, huge efforts have been made to understand the mechanisms involved in viral transmission, replication, and infection in an attempt to control the epidemic, protect against transmission, and cure those infected. Despite many prevention campaigns and the availability of medical devices, every year since 2010, around 2 million new infected cases have been counted.^1^ Progress in drug development produced highly active antiretroviral therapy (HAART), which has considerably improved life expectancy and quality of life for HIV-carriers.^2^ However, today, HAARTs alone are insufficient to control the epidemic because they fail to effectively eradicate the virus in treated individuals^3^ and they are only available to a limited number of patients.^1^ Today, all the epidemiological models predict that to efficiently control the spread of HIV would require efficient prophylactic strategies, like vaccines.

Until now, only three prophylactic vaccine candidates have completed the efficacy trials of phases II-b and III. The results of these trials were unexpectedly disappointing. AIDSVAX, which was based on the HIV envelope protein gp120 and an alum adjuvant, failed to effectively protect against HIV infection, even though it triggered the production of high levels of autologous neutralizing antibodies in humans.^4^ The subsequent STEP/PHAMBILI trials tested the protective capacity of three injections of adenovirus 5 vector delivering Gag, Pol, and Nef HIV antigens; this strategy resulted in an increased risk of HIV infection in vaccinated individuals with preexisting anti-adenovirus immunity.^5–7^ Finally, the RV144 trial combining AIDSVAX with a canarypox-based recombinant vector reduced HIV acquisition risk by about 31%. Although this effect is considered insufficient to impact the HIV epidemic, it significantly helped to unravel the immune-correlates of protection triggered by binding of IgG antibodies to variable regions 1 and 2 (V1V2) of gp120,^8,9^ as well as the contribution of CD4+ T-cell-specific responses^10^ and antibody-dependent cellular cytotoxicity (ADCC).^11,12^ However, although broadly neutralizing antibodies (bNAb) represent one of the most powerful approaches to control infection and block transmission in non-human primate (NHP) models, no vaccine has yet been produced which can elicit significant and sustained levels of bNAb.^13^ In addition, future vaccines should seek to induce cytotoxic CD8+ T-cell responses, to allow clearance of infected cells while healthy CD4+ T cells orchestrate appropriate immune responses.^14^

Particulate systems, including viral vectors and synthetic carriers, have proven to be excellent tools for the delivery of antigens to antigen-presenting cells (APC), promoting immune-specific responses with the production of antibodies and activation of cytotoxic T lymphocytes. Indeed, for their efficient capture and processing by APC, antigens must be in a particulate state. Many different types and sizes of vectors have been explored. Among them, synthetic vectors like polymeric particles and liposomes were compared to biological vectors like virus-like-particles (VLP).^15^ Despite the immunostimulatory properties of VLP, synthetic particles are usually preferable as they have a better safety profile and are easier and more cost-effective to manufacture.^16^ In particular, particles measuring less than 100 nm in diameter are of considerable interest as they can enter lymphatic vessels to access lymph nodes where they directly interact with APC such as dendritic cells (DC) and macrophages.^17,18^ Therefore, lipid particles are better than polymeric particles, as they are smaller in size. Further, the improved stability of nanostructured lipid carriers (NLC) in biological media compared to liposomes makes them highly attractive. Hence, NLC are an ideal system for the vectorization of synthetic vaccine antigens. To further promote antigen-associated immune responses, immunostimulatory molecules can be transported by these types of particulate systems, with the aim of delivering a “danger” signal to APC in combination with the antigen for optimal activation of these immune cells.^19^

We recently described the delivery of protein antigens to DC for efficient antigen presentation to T cells using nanostructured lipid carriers (NLC). Among their multiple advantages, NLC are highly stable over time and can interact with APC due to their small size associated with efficient lymphatic drainage. As a result, once administered to mice, NLC conjugated to the model antigen ovalbumin promoted both humoral and cellular immune responses.^20^ We previously reported a comparison of four NLC with different sizes and surface charges. This comparison allowed us to identify the prototype for vaccine formulation which produced the highest antibody titers and most extensive cellular responses. Here, we selected p24 as a relevant HIV antigen and to evaluate the efficacy of NLC for targeting it to DC. HIV-1 p24 has a highly conserved structure^21^ and has been widely evaluated as a vaccine immunogen in NHP studies.^22–29^ Indeed, anti-p24 cell-mediated responses were associated with viral control in HIV-infected patients.^30–32^ Furthermore, p24 represents a true challenge considering its poor immunogenicity,^28^ especially at inducing a humoral response. Therefore, we also analyzed p24-specific antibodies to assess the efficacy of our carrier at enhancing p24 humoral immunogenicity. Taking advantage of NLC’s versatility, we specifically designed particles bound with oligodeoxynucleotides (ODN) containing unmethylated cytosine–guanine repetitions (CpG) as an immunostimulant to be administered alongside p24 antigen. CpG is an adjuvant of great interest, known to induce an antiviral immunity with a pronounced Th1 profile.^33,34^ Moreover, it has been recently FDA-approved for a prophylactic vaccine for hepatitis B^35^ (Heplisav-B, Dynavax). In this study, we examined the immunogenicity of p24-bearing NLC in mice and non-human primates (NHP), as the most relevant animal model to assess anti-HIV immunity.^36,37^

## Results

**Fig. 1:**
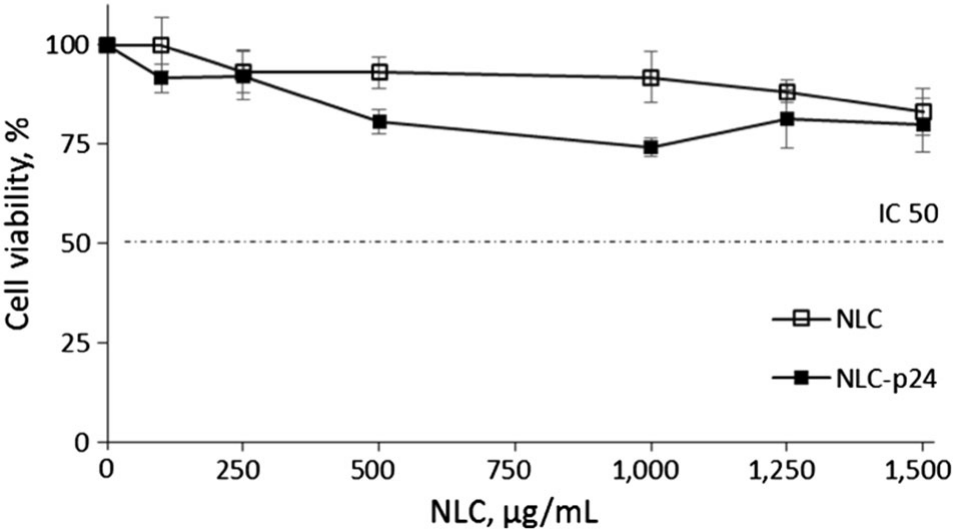
NLC formulations well tolerated by murine NIH/3T3 fibroblasts. Bars indicate the standard error of the mean calculated on three measurements

As our aim was to produce an effective HIV vaccine, we first determined the optimal conditions for vaccine antigen delivery. Gag p24 antigen was selected as a well-characterized HIV-1 capsid protein antigen which has been widely used in immunological studies involving NHP.^22–29^ Moreover, its highly conserved structure makes it suitable for use in a proof of concept approach for the design of a HIV vaccine.^21^ Ovalbumin was used in parallel to p24 as a control antigen. Either OVA or p24 proteins were bound to NLC (NLC–OVA and NLC–p24), with an optimized ratio of 50 proteins per nanoparticle, to allow multimeric presentation of the antigen. The hydrodynamic diameter of coated particles was around 80 nm, and they had a slightly anionic surface charge at neutral pH (Table 1). We have already demonstrated the safety of NLC–OVA;^20^ the safety of NLC–p24 was verified by assessing its cytotoxicity on murine NIH/3T3 fibroblasts (Fig. 1). Cells were exposed to doses of NLC–p24 ranging from 100 to 1500 µg/mL for 24 h. Dead cells were counted by flow cytometry after staining for surface expression of Annexin V and for membrane disruption using Propidium iodide. Results indicated that NLC–p24 were well tolerated by murine fibroblasts, with the IC50 not reached after exposure to the highest dose of 1500 µg/mL of particles.

**Table 1:** Physicochemical properties of antigen-grafted NLC

	Ratio (% w/w)	Ratio (nb Ag/NLC)	Hydrodynamic diameter (nm)	Polydispersity index	Zeta potential at pH 7.0 (mV)
NLC–OVA	1.28 ± 0.10	49 ± 4	83 ± 2	0.18 ± 0.02	−7 ± 1
NLC–p24	0.80 ± 0.06	50 ± 4	84 ± 2	0.18 ± 0.02	−5 ± 1

To perform immunogenicity studies, antigen formulations were combined with an immunostimulant, we compared CpG oligonucleotide to MPLA adjuvant. CpG is a TLR-9 agonist which has pro-inflammatory activity^38^ associated with the induction of humoral^39,40^ and cell-mediated immune responses^41,42^ through a Th1 polarization.^33,34^ In addition, CpG is a clinically relevant adjuvant, and has recently been approved by the FDA for use in a prophylactic vaccine for hepatitis B.^35^ MPLA is a synthetic analog of LPS with considerably reduced toxicity;^43,44^ as a result it has been approved for human use.^45^ It is reported to promote Th1^46^ and Th2^47^ polarization while also inducing potent humoral responses.^45^ These two adjuvants were co-administered with p24 antigen; results showed CpG to promote a 2-fold higher level of specific antibodies production compared to MPLA (Figure S1). In addition, CpG is much more convenient to use as MPLA presents solubility issues. Consequently, we used CpG in all subsequent immunogenicity studies.

**Fig. 2:**
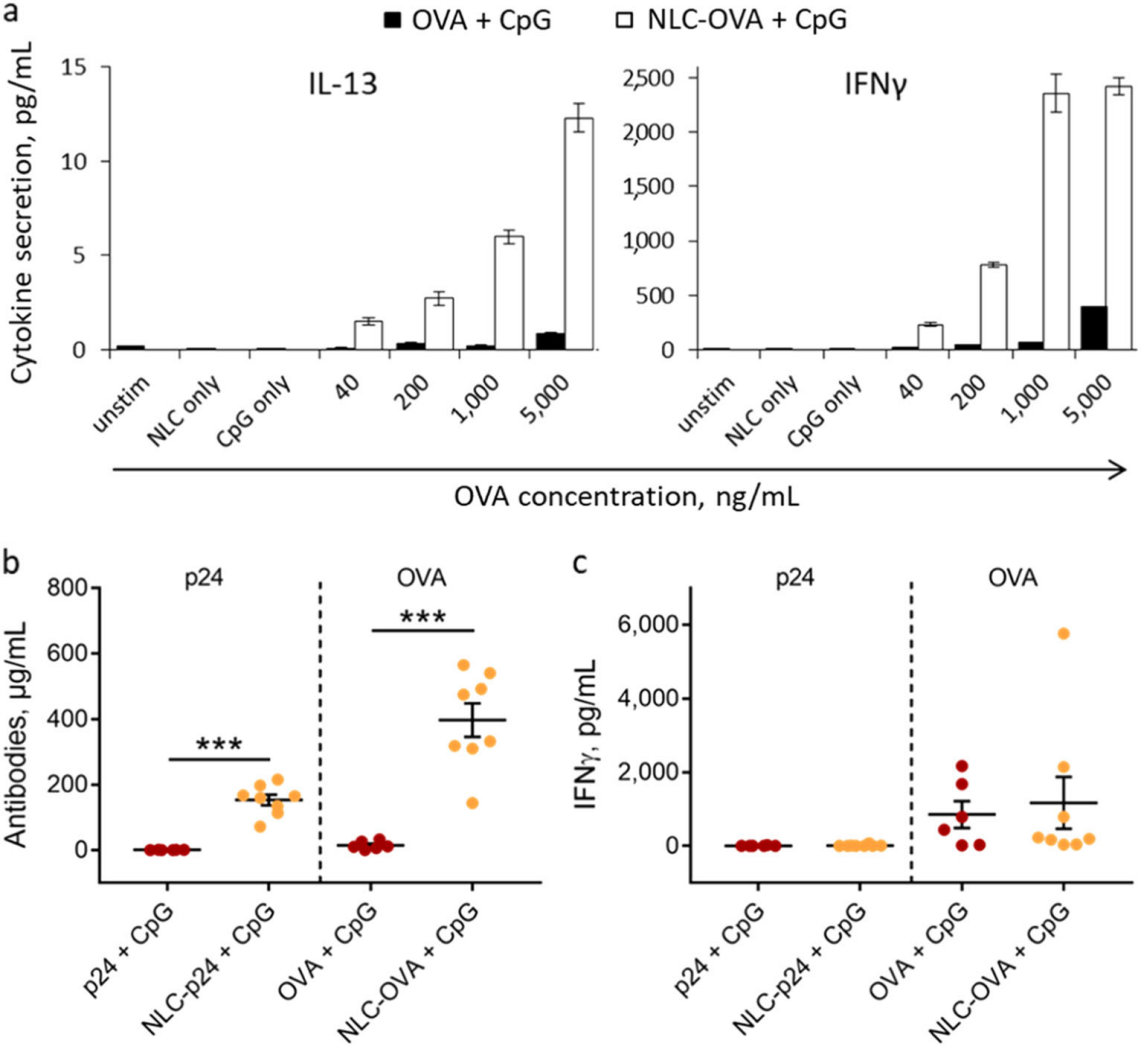
Immunogenic properties of NLC formulations. **a** Antigen presentation by primary DC to anti-OVA T lymphocytes. Secretion of IL-13 and IFN-γ by T lymphocytes after 4 days of co-culture with DC previously stimulated with formulations combining OVA and CpG. Bars indicate the standard error of the mean calculated on three measurements. **b** Humoral response in mice as measured based on serum anti-p24 and anti-OVA antibody levels and **c** cell-mediated response as determined based on the secretion of IFN-γ by T lymphocytes from the spleen of immunized mice after in vitro antigen stimulation. Mice were immunized with both p24 and OVA formulations (5 µg p24 and 10 µg OVA) co-administered with 10 µg CpG. Each point represents an individual mouse, horizontal bars represent the mean for the group and vertical bars indicate the standard error of the mean. Data were compared between groups using a 1-way ANOVA test followed by Fisher’s protected least significant difference test. ***p < 0.001

Having established the conditions for antigen-delivery to DC, we next examined how our system affected DC-mediated antigen presentation to T lymphocytes. To do so, NLC-OVA were compared to free OVA together with CpG. Primary DC were stimulated with NLC formulations for 5 h before mixing with OVA-specific T lymphocytes isolated from OT-II mice. After 4 days of culture, T lymphocyte activation was monitored by quantifying cytokine secretion in culture supernatants. IL-13 was chosen as an indicator of Th2 polarization, and IFN-γ for Th1 polarization. The results indicate that delivery of OVA by NLC significantly enhanced antigen presentation compared to free OVA. Indeed, secretion of both IL-13 and IFN-γ was 30-fold greater at an equivalent dose of 1000 ng/mL (“OVA + CpG”: 0.2 *±* 0.0 pg/mL IL-13 and 73 ± 1 pg/mL IFN-γ; “NLC–OVA+ CpG”: 6.0 ± 0.3 pg/mL IL-13 and 2357 ± 178 pg/mL IFN-γ; Fig. 2a). Controls with NLC and CpG alone confirmed that the response observed was indeed due to the OVA–NLC combination. These results demonstrate the advantages of delivering OVA antigen on NLC in combination with CpG-mediated immunostimulation, leading to enhanced Th2 and Th1 responses in vitro.

To assess whether NLC promotes p24-specific immune responses in a similar way, mice were immunized with both p24 and OVA formulations simultaneously to have an internal control to compare to p24 immunogenicity. Vectorized p24 was compared to free p24, both in combination with CpG immunostimulation. The humoral immune responses were evaluated by assaying specific antibodies in mouse serum. The production of p24-specific antibodies was significantly enhanced when p24 was delivered by NLC (“p24+ CpG”: 1 ± 0 µg/mL; “NLC-p24+ CpG”: 153 ± 16 µg/mL; *p* < 0.001), as was production of the control OVA-specific antibody (Fig. 2b). As a cell-mediated response is also highly desirable when designing a HIV vaccine, we also measured secretion of IFN-γ by T lymphocytes isolated from the spleens of immunized mice after in vitro stimulation with the appropriate antigens. Unexpectedly, in all cases, levels of IFN-γ were almost undetectable after p24 stimulation (Fig. 2c). In parallel, the OVA control produced highly heterogeneous values, and no significant difference was noted between vectorized and free OVA. Taken together, these results indicate that NLC potentially increases the intensity of the humoral response, but that it is not sufficient to induce IFN-γ secretion by activated T lymphocytes when associated with CpG as immunomodulatory compound. Therefore, our system required further optimization to efficiently trigger T-cell responses.

Given the advantages observed when NLC was used for antigen delivery, we applied the same concept for the vectorization of CpG with the aim of promoting better access to its intracellular receptor TLR-9, and as a result improving its adjuvant effect. CpG oligonucleotide is negatively-charged, thus can bind to cationic surfaces through electrostatic interactions. A new generation of cationic nanoparticles (NLC+) was designed to co-deliver the CpG adjuvant when mixed with antigen-presenting NLC (NLC–OVA and NLC–p24). NLC+ were obtained by adding cationic lipids to the formulation; a hydrodynamic diameter of 55 nm and a surface charge of +14 mV was measured for these particles in 154 mM NaCl at neutral pH. CpG spontaneously bound to NLC+ (NLC+ /CpG) when they were mixed together. When NLC+ was added in large excess, the complexation of CpG was complete and a ratio of around 20 CpG per particle was determined (Table 2).

**Table 2:** Physicochemical properties of cationic NLC formulations

	Ratio (% w/w)	Ratio (nb CpG/NLC)	Hydrodynamic diameter (nm)	Polydispersity index	Zeta potential at pH 7.0 (mV)
NLC+	0	0	55 ± 2	0.19 ± 0.02	14 ± 2
NLC+ /CpG	0.35 ± 0.02	20 ± 1	58 ± 2	0.19 ± 0.02	14 ± 2

**Fig. 3:**
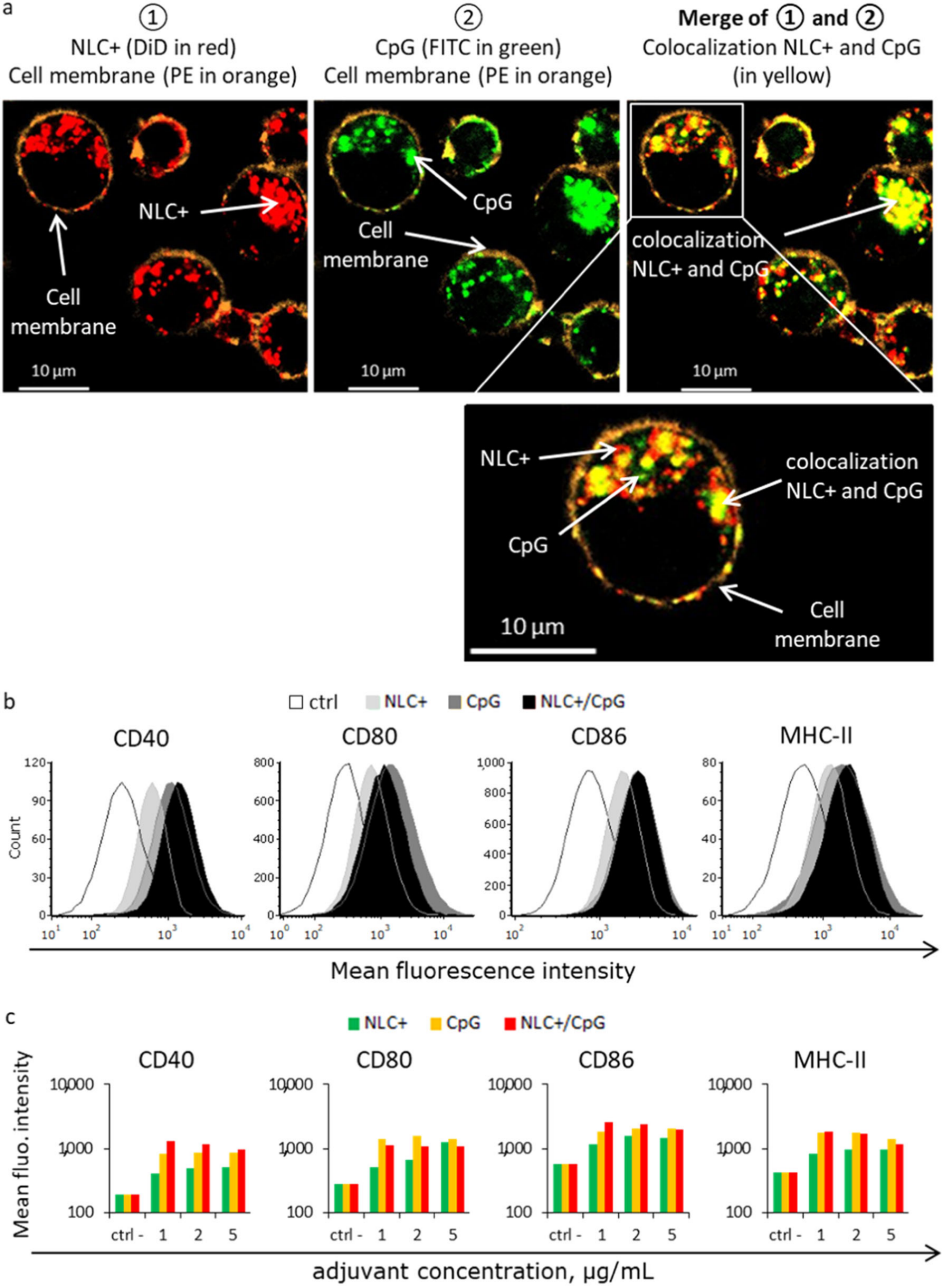
**a** Cellular localization of NLC+ /CpG after exposure of JAWS II DC for 1 h. Images were acquired by confocal microscopy. Red: NLC+ (DiD); green: CpG (FITC); orange: cell membrane (PE); Yellow: co-localization of NLC+ and CpG (merge of red and green). **b** Activation of DC by CpG formulations as measured based on the mean fluorescence intensity of activation markers CD40, CD80, CD86, and MHC-II after exposure to 2 µg/mL of CpG (and equivalent NLC+) and **c** 1, 2, 5 µg/mL of CpG (and equivalent NLC+) for 24 h

The impact of NLC+ complexation on transport of CpG inside APC was assessed by confocal microscopy. For this assessment, FITC-labeled CpG and DiD-loaded NLC+ were complexed together, incubated with JAWS II DC and then analyzed by confocal microscopy (Fig. 3a). After incubation for 1 h, NLC+ (in red) and CpG (in green) were captured and internalized into the cells, appearing within the cell membrane (in orange) (Fig. 3a: left panel in red, center panel in green). Interestingly, NLC+ and CpG were either found in their complexed form NLC+ /CpG, as indicated by co-localization (Fig. 3a: right panel yellow dots correspond to merging of red and green channels), or individuals forms (Fig. 3a: right panel red or green dots). These results confirmed that NLC+ and CpG were rapidly internalized together by DC and were subsequently separated inside the cells. This may promote efficient antigen presentation associated with substantial activation of intracellular TLR-9.

The impact of NLC+-mediated delivery of CpG on DC activation was determined by flow cytometry after exposure of JAWS II DC to free CpG, free NLC+ or NLC+/CpG complex for 24 h. For this purpose, four DC markers known to be upregulated upon activation were selected: the TNF receptor CD40, the co-stimulatory molecules CD80 and CD86, and MHC-II. Overexpression of all four markers was observed, indicating that all formulations could activate DC (Fig. 3b). However, NLC+ induced lower DC activation levels than CpG. The combination of NLC+ and CpG further enhanced the CpG-concentration dependent CD40 and CD86 expression, but no significant change was observed for the expression of MHC-II and CD80 *(*Fig. 3c*)*. As different adjuvant concentrations are known to increase or decrease cell activation depending on the immunostimulant used, the intermediate concentration of 2 µg/mL was selected for subsequent experiments. Altogether, these results highlighted the positive effects of NLC+ /CpG on DC activation, which is required to initiate a potent immune response.

**Fig. 4:**
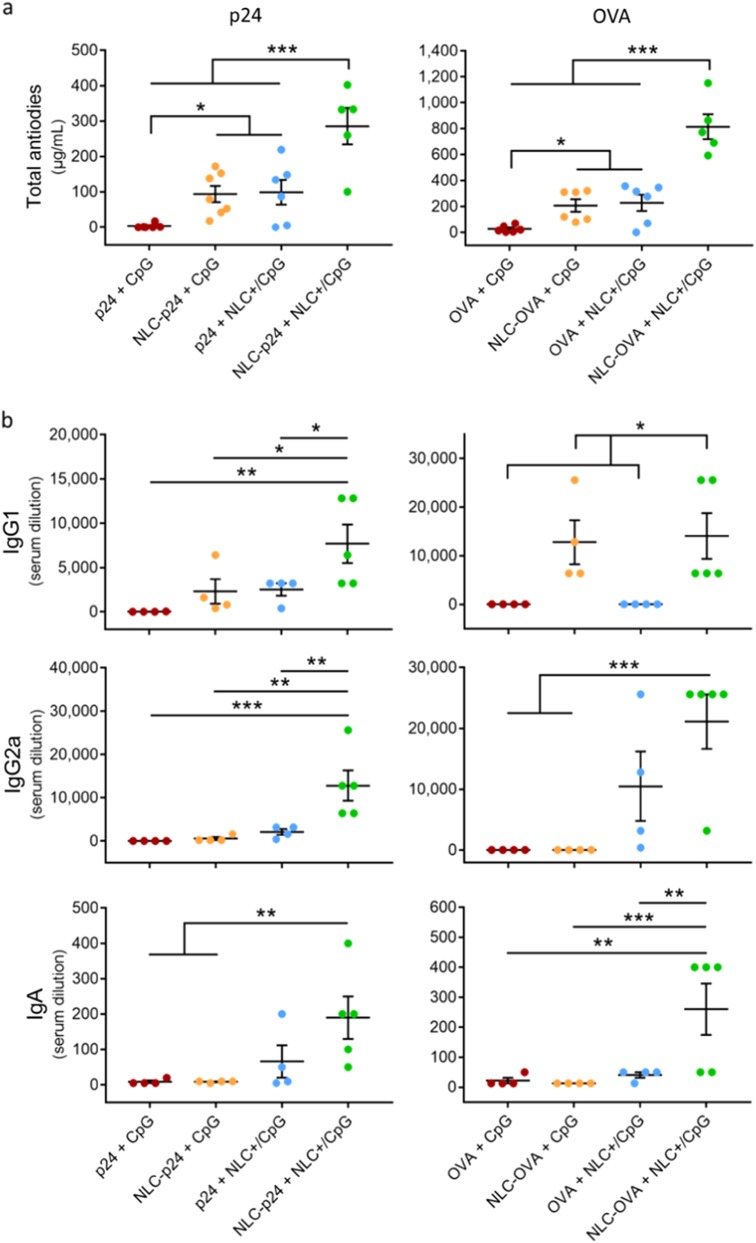
**a** Humoral response in mice following immunization with 5 µg of p24 and 10 µg of OVA in combination with 10 µg of CpG formulations, as measured based on total anti-p24 and anti-OVA antibody levels in muse serum. **b** IgG1, IgG2a, and IgA isotypes were also analyzed and are expressed as the highest serum dilution giving an absorbance of more than three times the background. Each point represents an individual mouse, horizontal bars represent the mean for the group and vertical bars indicate the standard error of the mean. Data were compared between groups using a 1-way ANOVA test followed by a Fisher’s protected least significant difference test. ***p < 0.001; **p < 0.01; *p < 0.05

The potency of these new particulate formulations was investigated in vivo by immunizing mice with different combinations of p24 and CpG, with OVA as internal positive control (Fig. 4a). As previously observed, vectorization of p24 by NLC enhanced the production of specific antibodies 30-fold (“p24+ CpG”: 3 ± 3 µg/mL; “NLC-p24+ CpG”: 94 ± 23 µg/mL; *p* = 0.042). Similarly, the delivery of CpG by NLC+ also increased antibody levels 30-fold (“p24+ NLC+ /CpG”: 99 ± 35 µg/mL; *p* = 0.038 compared to “p24+ CpG”). Co-delivery of p24 and CpG on distinct carriers further significantly improved the humoral response compared to all other groups (“NLC-p24+ NLC+ /CpG”: 285 ± 51 µg/mL; *p* < 0.001 compared to all groups). Similar trends were observed with OVA formulations, with the combination of vectorized OVA and CpG significantly increasing OVA-specific antibody titers, reaching 815 ± 96 µg/ml (*p* < 0.001 compared to all groups).

To provide further details on the quality of this humoral response, we used ELISA to determine the antibody isotypes produced, testing for IgG1, IgG2a, and IgA isotypes (Fig. 4b). IgG1 and IgG2a are commonly interpreted as correlates of Th2 and Th1 polarization, respectively. Serum IgA antibodies are involved in the inflammatory response and contribute to ADCC and phagocytosis by macrophages thanks to their high binding capacity.^48–50^ Using NLC to deliver p24 mainly promoted the production of IgG1 antibodies, whereas using NLC+ to vectorize CpG enhanced the production of isotypes IgG2a and IgA. However, the combination of both NLC for the co-delivery of p24 and CpG was the most potent formulation, significantly increasing production of all isotypes (“p24+ CpG” vs “NLC–p24+ NLC+ /CpG”; IgG1 *p* = 0.003; IgG2a *p* < 0.001; IgA *p* = 0.008). Although the IgG2a:IgG1 ratio only exceeded 1 in the case of the double vectorization of p24 and CpG, the difference was not statistically different compared to the other groups (Figure S2). The control with OVA showed a similar trend, with the combination of vectorized OVA and CpG promoting production of all isotypes most effectively (“OVA+ CpG” vs. “NLC–OVA+ NLC+ /CpG”; IgG1 *p* = 0.014; IgG2a *p* < 0.001; IgA *p* = 0.001). Together, these results support the versatility of NLC, capable of inducing humoral responses while also providing efficient antigen presentation and APC activation. These features are associated with intracellular delivery as a result of vectorizing both antigens, and the CpG-mediated immunostimulation. Indeed, the formulation containing vectorized antigens and immunostimulants was the most potent for both Th1 and Th2 polarization (IgG2a and IgG1) as well as IgA production. Isotype switching is known to be a T-helper-dependent mechanism, associated with increasing antigen-binding affinity.

**Fig. 5:**
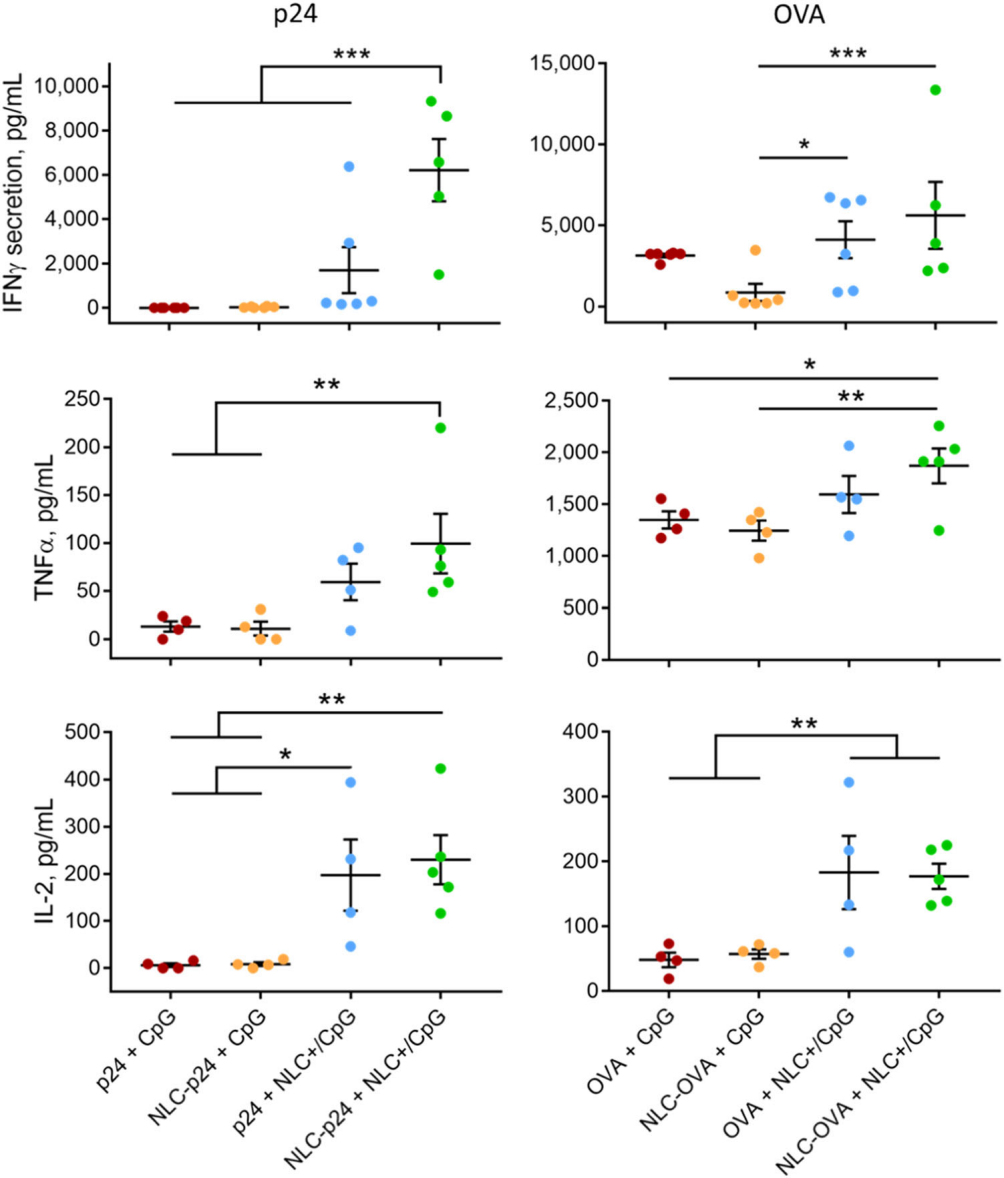
T-cell response after immunizing mice with 5 µg of p24 and 10 µg of OVA formulations in combination with 10 µg of CpG formulations, as measured based on IFN-γ, TNF-α, and IL-2 secretion by splenocytes after 72 h of antigen stimulation. Each point represents an individual mouse, horizontal bars represent the mean for the group and vertical bars indicate the standard error of the mean. Data were compared between groups using a 1-way ANOVA test followed by a Fisher’s protected least significant difference test. ***p < 0.001; **p < 0.01; *p < 0.05

Study of the cell-mediated immune response further highlighted the advantage of using the NLC+ carrier, as it potentiated IFN-γ, TNF-α, and IL-2 production by p24-stimulated splenocytes (Fig. 5). IL-2 is essential for the proliferation of T cells and is involved in the differentiation of naïve T cells into effector and memory T cells.^51^ TNF-α promotes inflammatory responses required for the recruitment of immune cells.^52^ IFN-γ is often measured as a marker for cell-mediated immune responses as it is produced by activated NK cells, CD4 Th1 and CD8 cytotoxic T cells.^53^ Our results showed minimal levels of all cytokines in the groups “p24+ CpG and “NLC–p24+ CpG” and increasing secretion in the groups “p24+ NLC+ /CpG” and “NLC–p24+ NLC+ /CpG”. In addition, the combination of vectorized p24 and CpG significantly intensified IFN-γ production compared to all other groups (“p24+ CpG”: 0 ± 0 pg/mL; “NLC–p24+ CpG”: 32 ± 11 pg/mL; “p24+ NLC+ /CpG”: 1696 ± 1037 pg/mL; “NLC–p24+ NLC+ /CpG”: 6220 ± 1403 pg/mL; *p* < 0.001 compared to all groups). The OVA control formulations confirmed that co-delivery of antigen and immunostimulant resulted in optimal T-cell stimulation, with up to 5619 ± 2067 pg/mL of IFN-γ produced.

**Fig. 6:**
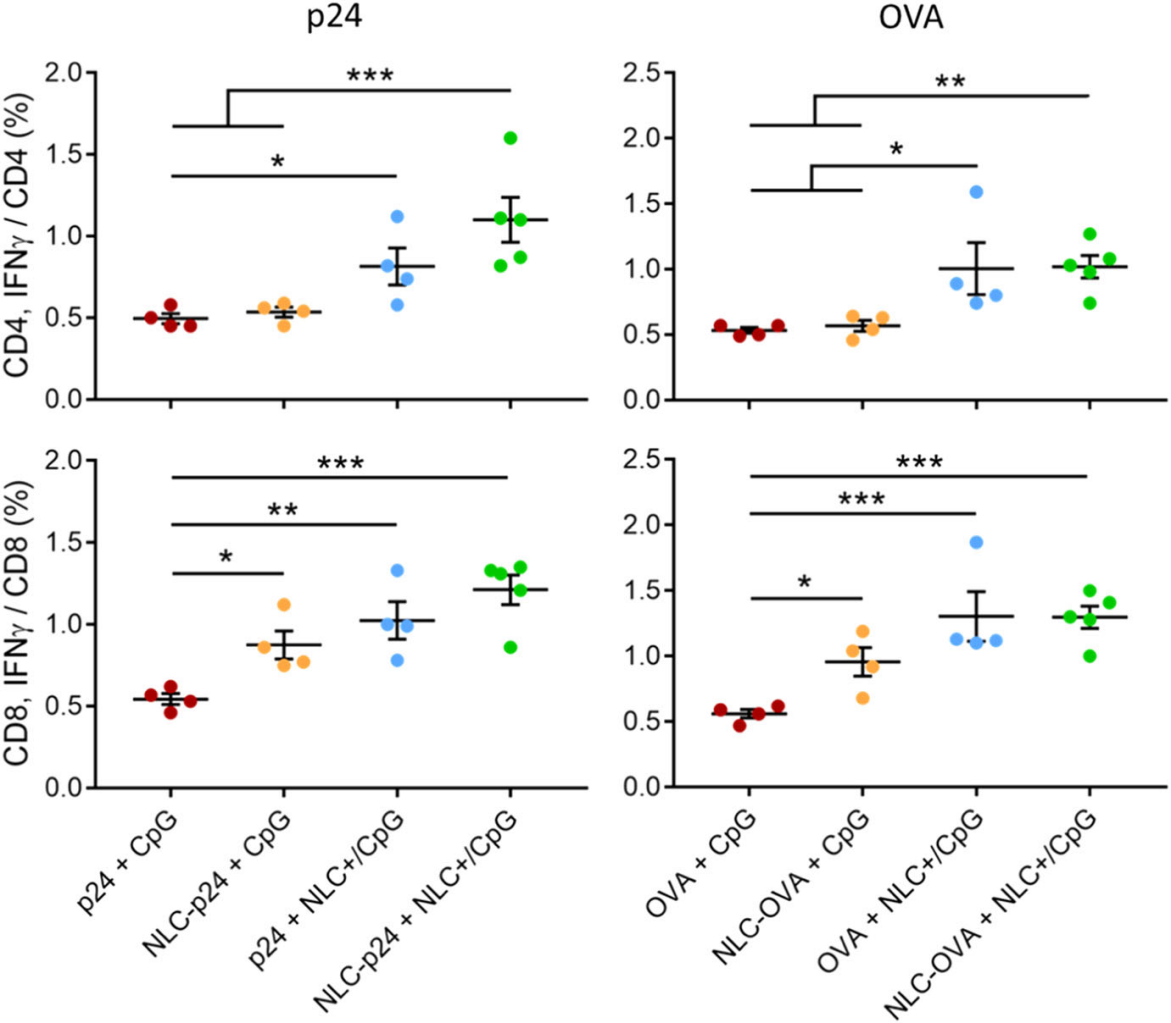
Antigen-specific CD4 and CD8 T-cell responses after immunization of mice with 5 µg of p24 and 10 µg of OVA co-administered with 10 µg of CpG formulations, as measured by intracellular staining (ICS). Splenocytes were stimulated with antigen for 24 h and then CD4 and CD8 T lymphocytes were stained intracellularly for IFN-γ and analyzed by flow cytometry. Each point represents an individual mouse, horizontal bars represent the mean for the group and vertical bars indicate the standard error of the mean. Data were compared between groups using a 1-way ANOVA test followed by Fisher’s protected least significant difference test. ***p < 0.001; **p < 0.01; *p < 0.05

Then, we performed intracellular staining (ICS) of antigen-stimulated splenocytes with the aim of determining CD4- and CD8-specific T-cell responses. Our results demonstrated that NLC vectorization affected both CD4 and CD8 T-cell activation, as these specific cells produced more IFN-γ when p24 and CpG were delivered by NLC (Fig. 6). Notably, using NLC to deliver p24 particularly stimulated CD8 T cells (“p24+ CpG”: 0.55 ± 0.03%; “NLC–p24+ CpG”: 0.88 ± 0.09%; *p* = 0.022) whereas NLC+ as CpG carrier activated both CD4 (“p24+ CpG”: 0.50 ± 0.03%; “p24+ NLC+ /CpG”: 0.82 ± 0.11%; *p* = 0.048) and CD8 T cells (“p24+ CpG”: 0.55 ± 0.03%; “p24+ NLC+ /CpG”: 1.03 ± 0.11%; *p* = 0.002). In addition, the use of both NLC for the delivery of p24 and CpG combined the advantages of each, promoting strong activation of both CD4 (“NLC–p24+ NLC+ /CpG”: 1.10 ± 0.14%; *p* < 0.001 compared to “p24+ CpG”) and CD8 T cells (“NLC–p24+ NLC+ /CpG”: 1.21 ± 0.09%; *p* < 0.001 compared to p24+ CpG). Interestingly, CD8 T lymphocytes produced significant amounts of intracellular IFN-γ when stimulated by “NLC–p24+ CpG” formulation, even though IFN-γ was not detected in splenocyte culture supernatants. We therefore hypothesized that CD8 T lymphocytes were partially activated following antigen presentation on MHC-I, triggering intracellular IFN-γ synthesis. However, the cytokine could have been retained inside the cell in the absence of a Th1 stimulation required for complete CD8 T-cell activation. Indeed, ICS showed that CD4 T cells stimulated by “NLC–p24+ CpG” did not produce IFN-γ. The OVA control further confirmed these immune orientations; co-delivery of OVA and CpG led to significantly higher activation of both CD4 (“OVA+ CpG”: 0.53 ± 0.02%; “NLC–OVA+ NLC+ /CpG”: 1.02 ± 0.09%; *p* = 0.006) and CD8 T lymphocytes (“OVA+ CpG”: 0.56 ± 0.03%; “NLC–OVA+ NLC+ /CpG”: 1.30 ± 0.08%; *p* < 0.001). These results suggest that immunization of mice with p24 and CpG vectorized by NLC not only induced activation of CD4 helper T lymphocytes, which orchestrate immune responses, but also CD8 T lymphocytes which are required for clearance of infected cells. These additional results underline the advantages of using NLC, the versatility of which allows the delivery of different antigens together with CpG immunostimulant to induce potent, and combined, immune responses.

**Fig. 7:**
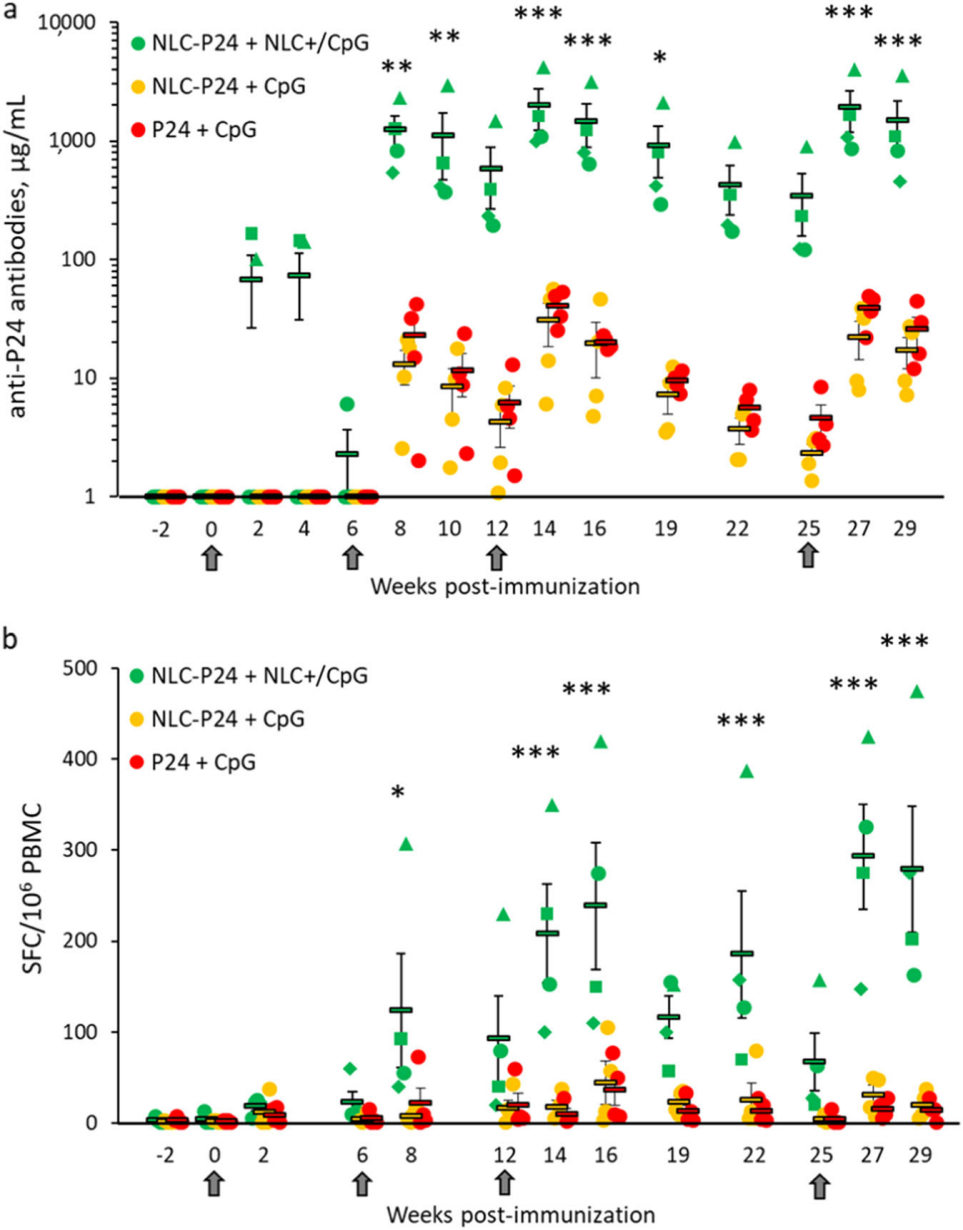
Immune responses in macaques (*N* = 4 per group) following four intradermal immunizations with NLC formulations containing the equivalent of 64 µg p24 and 253 µg CpG. **a** Specific antibodies and **b** IFN-γ-producing T lymphocytes. Individual macaques are represented by circular, triangular, square or diamond-shaped markers. Dashes represent the mean for each group, and bars indicate the standard error of the mean. Arrows below the X-axis indicate immunizations. Data were compared between groups base on a 2-way ANOVA test followed by Holm–Sidak method. ***p < 0.001; **p < 0.01; * p < 0.05 between NLC–p24+ NLC+ /CpG group and others

The results obtained in mice undoubtedly demonstrated the potency of NLC as a delivery system. To further investigate the performance of NLC, we next tested p24 formulations in NHP, using cynomolgus macaques as they are a relevant model for human immunity.^37^ Three groups of four animals immunized by the intradermal route with vectorized p24 and CpG (“NLC–p24+ NLC+ /CpG”), vectorized p24 plus free CpG (“NLC–p24+ CpG”), and free molecules (“p24+ CpG”) were compared. Analysis of p24-binding antibodies revealed that the group immunized with “NLC–p24+ NLC+ /CpG” produced significantly higher levels of immunoglobulins than the other groups, up to 1975 µg/mL on average and 4198 µg/mL for the best responder (Fig. 7a, *p* < 0.001 compared to all groups). Antibody production started from 2 weeks after priming (week 2) for 2/4 individuals from group “NLC–p24+ NLC+ /CpG” and was increased for 4/4 individuals from the first boost immunization (week 8). The next boost injection was followed by a 3-fold (week 14) increase in antibody levels, reaching a maximum concentration which was 50-fold higher than in the “p24+ CpG” group. The subsequent immunization was then delayed to allow study of how the p24-specific antibodies changed over time. A slowly decreasing humoral response was observed. However, antibody production remained significant up to 7 weeks after the third immunization, and the response remained detectable in all animals from group “NLC–p24+ NLC+ /CpG” at week 25. The aim of the fourth and last immunization was to verify that immune responses could be recalled even after a 3-month (13-week) lag. Maximum levels of immune responses were effectively recovered immediately after the last immunization (week 27). At the maximum, a 95-fold increase in the humoral response was measured compared to the “p24+ CpG” group at weeks 10, 12 and 19.

In addition to the humoral response, specific T-cell responses were assessed by ELISPOT for IFN-γ (Fig. 6b). Analysis of numbers of IFN-γ-producing cells over time showed a trend very similar to the antibody results. The main difference was that all groups responded, although with different magnitudes. From the first boost injection (week 8), the “NLC–p24+ NLC+ /CpG” group was significantly more efficient at activating T cells than the two other groups (*p* < 0.05 compared to all groups). Subsequent immunizations further increased the numbers of IFN-γ-producing T cells, particularly in the “NLC–p24+ NLC+ /CpG” group, with a 2-fold (week 14) and 4-fold (week 27) increasing up to a maximum of 293 SFC/10^6^ PBMC on average, and 475 SFC/10^6^ PBMC for the best responder (*p* < 0.001 compared to all groups). On weeks 14, 27, and 29, a 20-fold increase in the amount of IFN-γ-producing T cells was noted in the “NLC–p24+NLC+ /CpG” group compared to the “p24+ CpG” group. Interestingly, the same individual macaque from group “NLC–p24+ NLC+ /CpG” responded the best throughout the experiment in terms of p24-specific-antibody production and amount of IFN-γ producing cells (represented with triangles). Taken together, these results particularly highlighted the critical role of NLC+ in delivery of CpG alongside p24, as this was required to promote both humoral and T-cell immune responses. Indeed, no significant improvement was noted when only p24 was vectorized by NLC compared to free p24 and CpG. The benefits of using NLC include its versatility, which made it possible to successfully vectorize p24 antigen as well as CpG immunostimulant, leading to enhanced immune responses in NHP.

## Discussion

In this study, we examined the advantage of using NLC as a versatile vaccine delivery system to induce potent immune responses against HIV-1 p24 antigen, which is recognized as a poor immunogen.^28^ We chose to combine the antigen with CpG for its immunostimulatory properties,^33,34,39–42^ particularly antiviral immunity with a pronounced Th1 profile,^33,34^ and as a clinically relevant adjuvant recently approved for human use (Heplisav-B,Dynavax).^35^ In our previous study, we screened several NLC formulations and demonstrated that 80 nm NLC could promote humoral and cellular immune responses for the common antigen OVA co-administrated with the TLR-4 agonist LPS.^20^ We applied a similar concept here, using p24 and CpG, while keeping OVA as a positive control because of the poor immunogenicity of p24.^28^ Both antigens were systematically administered to the same mice. However, despite significantly increased antibody responses, the cellular immune responses obtained were very weak, even for OVA, suggesting weak immunostimulation by CpG. We hypothesized that by promoting CpG interaction with immune cells we could better trigger cell-mediated immunity. CpG is a ligand of intracellular TLR-9, and must therefore be internalized by DC to trigger its receptor. Receptor triggering is essential to enhance vaccine efficiency.^54^ Previous studies reported good results when immunostimulants were delivered using particulate systems.^19,55,56^ As CpG is a polyanionic oligonucleotide, we designed a cationic NLC to allow its vectorization. Although several studies have described the co-delivery of antigens and immunostimulants in the same particles, Mohsen et al.^57^ recently demonstrated that they can also be effectively processed by the same APC when carried by different particles, resulting in enhanced immune responses.

Here, we show that CpG vectorization by NLC+ not only enhanced antibody production, but also induced significant T-cell activation. Indeed, a 30-fold increase in p24-specific antibodies due to CpG delivery by NLC+ was observed when comparing groups of mice immunized with vectorized p24, reaching 285 ± 51 µg/mL. More importantly, NLC+ strikingly promoted IFN-γ secretion by p24-specific T cells, up to 6220 ± 1403 pg/mL, which is 194-fold higher than the levels induced when using free CpG. Previous studies mentioned similar trends when carriers for both antigens and immunostimulants were used, mostly working with the OVA model. Zhang et al.^19^ improved antibody titers up to 5-fold by co-delivering OVA and imiquimod in PLGA particles. IFN-γ production was also enhanced in their system, up to 200 pg/mL on average. Bal et al.^55^ described cationic liposomes for the co-delivery of OVA and CpG, which promoted the production of IgG2a antibodies and increased the secretion of IFN-γ up to 1000 pg/mL on average. Similarly, Ilyinskii et al.^56^ designed polymeric particles to carry OVA together with resiquimod or CpG in the same particle or in distinct ones, resulting in enhanced immune responses whatever the delivery strategy. Here, we report the preparation and use of nanoparticles to deliver both p24 and an immunostimulant and inducing high T-cell response. Importantly, in mice immunized with “NLC–p24+ NLC+ /CpG”, high levels of antigen-specific antibodies of different subclasses (IgG1, IgG2a, and IgA) were measured in their serum. Isotype switching is known to be a T-helper-dependent mechanism, associated with increasing antigen-binding affinity. Vectorization of antigen by NLC mainly promoted the production of IgG1 isotypes, while NLC+-mediated CpG delivery triggered IgG2a and IgA production. Increasing production of IgG2a isotype is associated with a Th1 polarization, required to promote cytotoxic T lymphocytes activity. In addition, serum IgA antibodies are involved in the inflammatory response and take part in ADCC and phagocytosis by macrophages thanks to their high binding ability.^48–50^ Using NLC also increased the production of IFN-γ by antigen-specific T lymphocyte subsets. Once again, distinct contributions were observed depending on whether NLC was used for antigen or immunostimulant delivery. Activation of CD4 T lymphocytes was mainly a consequence of CpG delivery by NLC+, while both NLC for antigen and CpG vectorization contributed to CD8 T lymphocyte activation. As they orchestrate immune responses, helper CD4 T lymphocytes are key actors in effective immune responses. Activation of CD8 T lymphocytes by NLC formulations is also highly significant as they are the main effectors of the cytotoxic response needed to clear infected cells. The use of NLC is therefore highly valuable considering the impact on the resulting cellular activation. Altogether, these results demonstrated the advantages of using NLC to deliver both antigen and immunostimulant in order to combine the benefits from each of them to induce complex and potent immune responses.

The best animal model for studying HIV remains NHP as they are very similar to humans, in particular with regard to their immune system.^36,37,58,59^ We thus applied our new particulate system for p24 and CpG delivery during macaque immunization. After a 7-month study comprising four intradermal immunizations and bi-monthly sampling to analyze immune markers, our results confirmed the huge potential of NLC for promoting both p24-specific humoral and cell-mediated immune responses. Strikingly, the group “NLC–p24+ NLC+ /CpG” was the only one to produce significantly higher p24-specific antibodies compared to “p24+ CpG” control group. Responses were detectable from just 2 weeks after priming for 2/4 animals, and all 4/4 macaques responded after the first boost immunization. Each additional immunization increased the humoral response, with antibody levels up to 1975 µg/mL measured on average and 4198 µg/mL for the best responder. The absence of improvement of the humoral response in the group “NLC–p24+ CpG” compared to the control group “p24+ CpG” was unexpected, considering the results obtained in mice. However, immunogenicity may be quite different in mice compared to NHP and humans. It has been described that immune responses to vaccines in NHP are similar to those in humans, contrarily to the mouse model.^37,59,60^ This justifies the use of NHP to evaluate vaccine strategies which look promising in mice.^60^ Furthermore, varying immunization protocols have been used in mice and NHP, in terms of number of injections, p24 and CpG doses, administration routes as well as CpG batches, thus likely explaining these specie differences. Indeed, mice were immunized with ODN 1826, a murine class B CpG known to highly activate B cells, whereas NHP were immunized with ODN M362, a human class C CpG known to stimulate pDC and B cells. The administration route is also known to impact immune responses.^61^ Despite these differences, results from mice and NHP studies have in common that the group “NLC–p24+ NLC+ /CpG” yielded the highest antibody titers. Although p24-specific IFN-γ-producing cells were induced with all formulations from the first boost injection, the magnitude of the response was 20-fold higher in the group, which received NLP-delivered p24 and CpG. Maximal numbers of IFN-γ-producing cells were recorded as 293 SFC/10^6^ PBMC on average, and 475 SFC/10^6^ PBMC for the best responder. These humoral and cellular immune responses remained detectable after 13 weeks without stimulation and reached their maximum with the final immunization.

To our knowledge, the description of high magnitude p24-specific immune responses in NHP has rarely, if ever, been reported with synthetic carriers. Importantly, the immune responses induced here were comparable to those achieved with viral vectors.^29,62,63^ However, at an equivalent order of magnitude, our system presents relevant advantages over viral vectors, such as a much better safety profile and a more straightforward manufacturing process. Only very few studies have been conducted on NHP to assess HIV-1 p24 immunogenicity using synthetic vectors. Among these, Ataman-Önal^22^ reported anionic PLA (poly-lactic acid) particles of 500 nm in diameter coated with p24. In their immunogenicity studies, 2 macaques received a high dose of 500 µg of p24, and enhanced antibody production (maximal serum dilution 1,000,000) and numbers of IFN-γ-producing cells (700 spots/10^6^ PBMC) were measured in 1/2 good responder. In another study, the immunogenicity of p24 was strengthened by fusion with an antibody targeting the DEC-205 receptor on DC (DEC-p24), which is known to promote antigen cross-presentation.^23^ In combination with a high dose of Poly IC:LC (1 mg), DEC-p24 (60 µg of p24) immunizations induced a potent cellular response in NHP, with 1300 SFC/10^6^ PBMC on average. A similar strategy was reported by Flamar et al.,^27^ who described the fusion of p24 with an antibody targeting an immunoreceptor on DC. The immunogenicity of 60 µg p24 was examined with or without adjuvant (250 µg poly I:C); a maximum 6-fold increase in antibody production was reported. Although the results from these studies remain difficult to compare due to differences in several key parameters (prime/boost protocol, antigen and immunostimulant doses, routes of administration, etc.), our results clearly exceed previously reported levels of antibody secretion and numbers of IFN-γ producing cells in response to rational doses of p24 (64 µg) and CpG (253 µg). Moreover, we also present results of monitoring of NHP immune markers every 2 to 3 weeks over 7 months, and demonstrate potent immune responses specific to the poorly immunogenic HIV-1 p24 antigen.

**Fig. 8:**
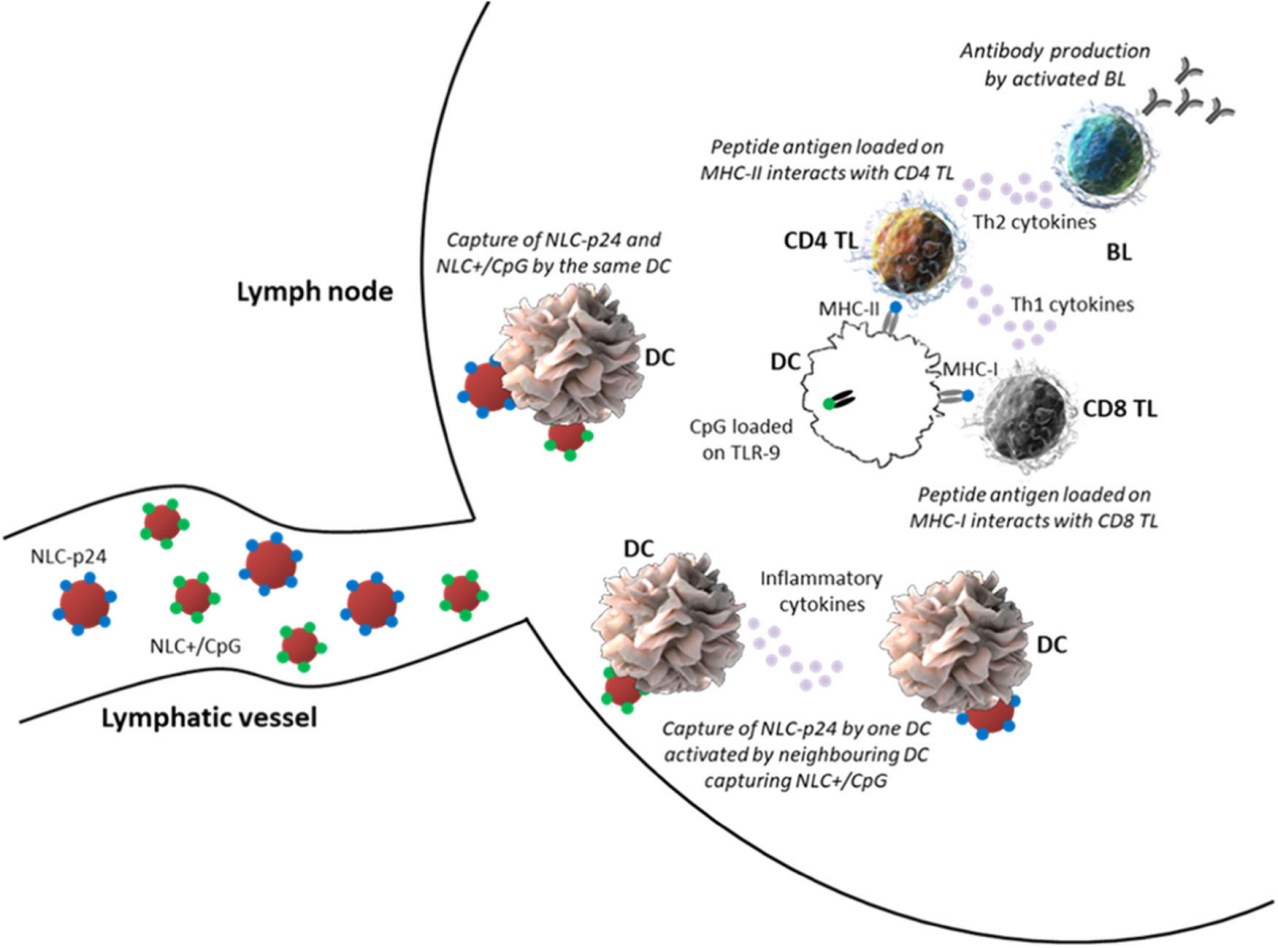
Graphical representation of NLC interactions with immune cells in lymph nodes. Within lymph nodes, DC would be activated by the capture of both NLC–p24 and NLC+ /CpG or by inflammatory cytokines produced by neighboring DC having captured NLC+ /CpG. Depending on p24 processing, antigenic peptides will be loaded on MHC-II or MHC-I for presentation to CD4 or CD8 T lymphocytes, respectively. Specific antibodies will be produced by B cells stimulated by Th2 cells, while CD8 T lymphocytes will differentiate into CTL following stimulation by Th1 cells. DC dendritic cell, TL T lymphocyte, BL B lymphocyte. Image credit: for the dendritic cell image, Donald Bliss, National Library of Medicine (source: Sriram Subramaniam, National Cancer Institute, 2010);^72^ for the T and B lymphocyte images, Blausen.com staff (2014), WikiJournal of Medicine 1 (2)^73^

After being injected in vivo, NLC loaded with antigens or CpG are able to circulate through lymphatic vessels due to their small size ranging below 150 nm, as evidenced in our previous studies with naked NLC^64^ or NLC bearing protein antigens.^20^ When reaching lymph nodes, NLC will interact with local APC, especially DC. Two scenarios are possible. In the first case, both NLC–p24 and NLC+ /CpG would be captured by the same APC, leading to its activation. In the second case, one APC would capture NLC–p24 and then be activated by inflammatory cytokines coming from a neighboring APC, being itself activated following the capture of NLC+ /CpG. The resulting activated APC will load antigenic peptides on MHC-II for presentation to CD4 T lymphocytes. B cells will be stimulated by a Th2 polarization, leading to the production of p24-specific antibodies. In some cases, NLC p24 would escape the endosome and antigenic peptides would be loaded on MHC-I for presentation to CD8 T lymphocytes. These cells would be fully activated with a Th1 stimulation, leading to their differentiation into p24-specific cytotoxic T lymphocytes (CTL) (Fig. 8).

As a fully synthetic platform, NLC has numerous advantages over whole attenuated or killed inactivated pathogens as well as viral vectors. First, they are much safer as they are non-infectious and therefore do not require any assessment of viral clearance during their manufacturing process. As a result, scale-up is less troublesome and industrial transfer can be more simply and cost-effectively achieved. In addition, NLC are highly stable and easy to store and transport, and do not require a temperature-controlled environment. In our previous study we showed that NLC could rapidly reach lymph nodes by lymphatic drainage, thus better targeting immune cells for antigen delivery, which is highly relevant in a dose-sparing strategy. In the present study, we tested an efficient co-delivery approach, using as many vectors as there were biomolecules to deliver. This method could therefore be extended to any additional antigen or immunostimulant needed and is appropriate to precision medicine where the dose of each component can be adjusted as required. Finally, we demonstrated the use of NLC to deliver different biomolecules, namely OVA and p24 antigens as well as CpG immunostimulant, this approach could potentially be extended to other pathogens as a universal platform for vaccines. Finally, the NLC technology could also be further combined with other delivery technologies, such as dermal patches or microneedles, thus allowing the design of vaccine administration through a needle-free approach.^65^

In terms of perspectives, NLC can be associated with other rational HIV antigens, such as envelope glycoproteins gp120 or gp41. With such a multi-antigen vaccine formulation, we aim at inducing multi-targeted immune responses, namely Env-specific broadly neutralizing antibodies and p24-specific cytotoxic responses, for protection against HIV.

## Methods

### NLC synthesis

#### Preparation of basic NLC

NLC were prepared as described elsewhere.^66^ Briefly, a lipid phase containing triglycerides (Suppocire NC^TM^, Gattefossé; super refined soybean oil, Croda Uniqema) and phospholipids (Lipoid S75; Lipoid) and an aqueous phase containing PEGylated surfactants (Myrj^TM^ S40, Croda Uniqema) were prepared separately before being mixing for ultrasonication. The resulting NLC were purified by dialysis against PBS (MWCO: 12–14 kDa, ZelluTrans). Three different batches were formulated by applying this general procedure, either the composition of the lipid phase or the aqueous phase was adjusted to produce the following NLC types: NLC–SH for OVA grafting, NLC–mal for p24 grafting and cationic NLC (NLC+). All final particles were filtered on a 0.22 µm cellulose membrane (Millipore) and characterized using a Zeta Sizer Nano ZS (Malvern Instrument) to determine their hydrodynamic diameter and zeta potential based on dynamic light scattering (DLS) and electrophoretic light scattering (ELS), respectively. In addition, all batches of NLC were subjected to cytotoxicity experiments to validate their safety profile.

#### Preparation of NLC–OVA via NLC–SH

OVA-grafted NLC (NLC–OVA) were obtained as previously reported.^20^ Thiol-functionalized NLC (NLC–SH) were prepared by adding 5 mg of (2-pyridildithio) propionate-functionalized PEGylated surfactants to the aqueous phase and by reducing the disulfide bond using TCEP (tris(2-carboxyethyl) phosphine hydrochloride, Sigma Aldrich). The resulting NLC–SH were purified by dialysis and filtered on a 0.22 µm membrane. Functional maleimide groups were introduced on OVA (OVA–mal) by derivatization of 1 mg OVA (Bio Basic Inc.) with 200 µg sulfo-SMCC (sulfosuccinimidyl 4-(*N*-maleimidomethyl)cyclohexane-1-carboxylate, Thermo Scientific). Excess linker was removed by gel filtration on Sephadex G25 gel in a PD10 desalting column (GE Healthcare). OVA–mal (1 mg) was added dropwise to a solution of 17 mg NLC–SH, under gentle stirring at 4 °C. The reaction was allowed to proceed overnight at room temperature. Free thiols were capped using *N*-(2-hydroxyethyl)maleimide (Sigma Aldrich). NLC–OVA were then purified by size exclusion chromatography (SEC) on Superdex 200 gel (GE Healthcare). To determine the number of OVA per NLC, the same reaction was performed in parallel using fluorescently-labeled moieties: DiD-loaded NLC–SH and fluorescein-tagged OVA, and fluorescence titration was performed (both dyes from Invitrogen).

#### Preparation of NLC–p24 via NLC–mal

For p24 grafting, maleimide-presenting NLC (NLC–mal) was prepared by adding 5 mg of (*N*-maleimidomethyl) cyclohexane-functionalized PEGylated surfactants to the aqueous phase; NLC–mal were then purified by dialysis and filtered on a 0.22 µm membrane. Thiol groups were introduced on p24 (p24–SH) by derivatization of 1 mg p24 (PX’Therapeutics) with 10 µg of 2-iminothiolane (Thermo Scientific). Excess linker was removed by gel filtration as for NLC–SH. p24–SH (1 mg) was added dropwise to a solution of 30 mg NLC–mal. Free maleimide functions were capped with 2-mercaptoethanol (Sigma Aldrich) and the resulting NLC–p24 was purified by SEC. Fluorescence titration was performed using DiD-loaded NLC–mal and fluorescein-tagged p24.

#### Preparation of NLC+

Cationic NLC (NLC+) were prepared by adding cationic and fusogenic surfactants (Avanti Polar Lipids) to the lipid phase, i.e., DOTAP (1,2-dioleoyl-3-trimethylammonium-propane chloride) and DOPE (1,2-dioleoyl-sn-glycero-3-phosphoethanolamine), respectively. Complexation of NLC+ with negatively-charged CpG oligodeoxynucleotides (ODN1826 for mice and M362 for macaques, Invivogen) occurred spontaneously upon mixing with an amine groups to phosphate groups ratio of 48. Binding was verified by electrophoretic mobility shift assay.

### Cells

To ensure reproducibility, which can be difficult to guarantee with primary cells, the murine dendritic cell line JAWS II (ATCC) was used in all in vitro assays. Primary dendritic cells (DC) and T lymphocytes were used for antigen presentation experiments. Primary DC were prepared from C57BL/6 mice femurs as previously described.^67,68^ Briefly, femurs were collected and flushed with Iscove’s modified Dulbecco’s medium (IMDM) to harvest cells. Erythrocytes were removed by negative magnetic cell sorting after labeling with biotinylated TER-119 and Ly-6G/Ly-6C antibodies together with Dynabeads biotin binder (Invitrogen). Progenitor cells were grown for 10 days in complete IMDM supplemented with GM-CSF, FLT-3L and IL-6 (Invitrogen) to produce fully active DC. The phenotype of the resulting DC was verified by analyzing the expression of differentiation markers CD11b and CD11c by flow cytometry on an LSR II flow cytometer (BD Biosciences). Primary T lymphocytes were purified from OT-II mouse spleens. Briefly, the spleen was collected and splenocytes harvested by mechanical grinding on a 100 µm cell strainer. Erythrocytes were eliminated using RBC lysis buffer. T lymphocytes were isolated by negative magnetic sorting using Dynabeads Untouched Mouse T cells kit (ThermoFisher Scientific), according to the manufacturer’s instructions.

### Cytotoxicity

Cytotoxicity of NLC formulations was assessed on NIH/3T3 fibroblasts using Dead Cell Apoptosis Kit (ThermoFisher) and flow cytometry. Briefly, 10^6^ cells/mL were exposed to NLC formulations for 24 h. Cells were then stained with anti-Annexin V-FITC antibody and Propidium Iodide according to the kit instructions. Apoptotic and dead cells were detected by flow cytometry. Results are expressed as a percentage of live cells after subtraction of the staining level measured for the negative control, corresponding to non-exposed cells. Samples were analyzed in triplicate.

### Confocal microscopy

JAWS II DC were seeded at 10^6^ cells/mL in 24-well plates and incubated overnight at 37 °C. NLC+ /CpG were prepared by complexation of DiD-labeled NLC and FITC-labeled CpG (Invivogen). Labeled NLC (100 µg/mL) were added to the wells and incubated for 1 h. Then, cells were washed in PBS and allowed to adhere to poly-L-lysine-coated slides for 1 h at room temperature. Cells were fixed in paraformaldehyde 4% for 10 min and washed in PBS. Plasma membrane was labeled using cholera toxin–biotin revealed by streptavidin-PE for 30 min at room temperature. After the final wash, slides were mounted in Dako mounting medium and stored overnight in the dark at room temperature. Images were acquired on a ConfoCor 2 microscope (Zeiss) using LSM 510 software. Imaged were treated using ImageJ software. Slides were stored long-term at −20 °C.

### Cellular activation

JAWS II DC were seeded at 10^6^ cells/mL in 24-well plates and incubated overnight at 37 °C. Immunostimulatory formulations containing CpG and NLC+ were added to the wells, at 1, 2 or 5 µg/mL of CpG. After 24 h, cells were washed in PBS and stained with fluorescent antibodies (BioLegend) for MHC-II, CD40, CD80, and CD86 expression for 30 min at 4 °C in PBS + 1% BSA. Stained cells were washed before analysis by flow cytometry.

### Antigen presentation

Primary DC, used as APC, were seeded at 2 × 10^5^ cells/mL in 24-well plates and incubated overnight at 37 °C. NLC formulations containing OVA and CpG were added to the corresponding wells and incubated for 5 h. Specific anti-OVA T lymphocytes were purified from OT-II mice, mixed with DC at 8 × 10^5^ cells/mL and co-cultured for 4 days. Each condition was performed in duplicate. Supernatants were collected to determine IL-13 and IFN-γ levels using ELISA Ready-Set-Go kits (eBiosciences) according to the manufacturer’s instructions.

### Animals and ethics statement

BALB/c and C57BL/6 female mice were purchased from Janvier. They were housed in the animal facility of the Institute for Advanced Biosciences (France) according to institutional guidelines. The animal experimentation protocol was approved by the local ethics committee (ref. 2015062612254918). No randomization of animals was performed.

Adult cynomolgus macaques (Macaca fascicularis) were imported from Mauritius and housed in the facilities of the Infectious Disease Models and Innovative Therapies (IDMIT) Center (CEA, Fontenay-aux-Roses, France). Both male and female animals were included, with a 1:1 sex-ratio in each experimental group. NHPs were used at the CEA in accordance with French regulations and under the supervision of national veterinary inspectors (CEA Permit Number A 92-032-02). The CEA complies with the Standards for Human Care and Use of Laboratory Animals, as set out by the Office for Laboratory Animal Welfare (OLAW, USA) and is accredited under OLAW Assurance number #A5826-01. The use of NHP at the CEA complies with the recommendations in European Directive 2010/63 (recommendation N°9). The animals were used under the supervision of the veterinarians responsible for the animal facility. This study was approved and accredited under statement number A15-085 by the ethics committee “Comité d’Ethique en Expérimentation Animale du CEA”, registration number 44 for the French Ministry of Research. Animals were housed in pairs in modules allowing social interaction, under controlled humidity, temperature and light conditions (12 h light/12 h dark cycles). Water was available ad libitum. Animals were monitored and fed 1–2 times daily with commercial monkey chow and fruit by trained personnel. Macaques were provided with environmental stimuli including toys, foodstuffs and music under the supervision of the CEA’s Animal Welfare Body. Experimental procedures (animal handling, immunization protocols and blood sampling) were conducted after sedating animals with ketamine chlorhydrate (Rhône-Mérieux, Lyon, France, 10 mg/kg).

For both mice and NHP experiments, investigators were not blinded to the group allocation.

### Immunizations and sampling

On day 0, groups of 4–8 of 8-week old female BALB/c mice were immunized intra-peritoneally with an initial injection containing the equivalent of 10 µg OVA or 5 µg p24, either in free form or grafted to NLC. The size of the groups for each experiment was set in accordance to ethic comity recommendations in order to get enough power for statistics and to follow the “3 R rules” aiming to reduce animal suffer. The two antigens were co-administered with 10 µg CpG. On day 21, a booster shot was administered in the same conditions. On day 28, blood samples were collected from the vena cava using a 25 G needle, and spleens were harvested.

Groups of 4 macaques were immunized on weeks 0, 6, 12, and 25 by intradermal injection with 64 µg p24, either in free form or grafted to NLC. Antigen was co-administered with 253 µg CpG, either in free form or associated with NLC. Blood samples were collected before and after each immunization to analyze the specific serum-antibody response and the cellular response in peripheral blood mononuclear cells (PBMC).

### Antibody assays

Anti-OVA and anti-p24 antibodies were measured in mouse sera by ELISA. Briefly, 96-well microplates were coated with 1 µg/mL antigen (OVA or p24) and stored overnight at 4 °C. A PBS blocking solution containing 1% BSA was used to prevent non-specific adsorption. Diluted murine sera were applied to the wells for 2 h at room temperature under gentle agitation. Subsequently, an anti-mouse-HRP antibody (Sigma Aldrich) was added for 1 h. Binding was revealed by TMB enzymatic reaction (BD Biosciences), the reaction was stopped after 15 min by adding sulfuric acid. Absorbance was read at 450 nm on a VICTOR microplate reader (Perkin Elmer). Standard curves were produced using serial dilutions of monoclonal mouse anti-OVA (Enzo Life Sciences) and anti-p24 (Abcam) antibodies, and used to express antibody titers in immunized mice. IgG1, IgG2a, and IgA isotypes were determined using anti-mouse IgG1-HRP (ThermoFisher), anti-mouse IgG2a-HRP (ThermoFisher), and anti-mouse IgA (Novus Biologicals) detection antibodies. As no isotype standard antibodies were used, serial dilutions of sera were performed to determine the highest dilution giving an absorbance greater than 3 times the background. The same protocol was used to measure antibodies in macaque sera, using an anti-human-HRP antibody for detection which cross-reacts with macaque antibodies according to the manufacturer (MyBioSource, MBS8225067) and a monoclonal human anti-p24 antibody (MyBioSource, MBS588073) to produce a comparative standard curve.

### T lymphocyte assays

Fresh mouse splenocyte suspensions were prepared from spleens by mechanical grinding on a 100 µm cell strainer; erythrocytes were removed using RBC lysis buffer. Cells were stimulated for 3 days with 50 µg/mL OVA or 10 µg/mL p24 in RPMI-1640 complete culture medium supplemented with 10% FCS (Life Technologies), 1% penicillin/streptomycin (Invitrogen), 1% non-essential amino acids (Life Technologies) and 1% sodium pyruvate (Life Technologies). Supernatants were collected to measure IFN-γ secretion using mouse IFN-γ ELISA Ready-Set-Go (eBiosciences).

Specific T-cell responses were analyzed in macaques by IFN-γ ELISPOT assay^69–71^ (Monkey IFN-γ ELISpot PRO kit, Mabtech). Macaque PBMC were isolated from whole blood using vacutainer sodium heparin CPT tubes (BD Biosciences). PBMC were stimulated for 18 h with a pool of overlapping 15-mer peptides covering the entire sequence of HIV p24. Spots were counted with an Automated ELISpot Reader ELR08IFL (Autoimmun Diagnostika GmbH).

### Intracellular IFN-γ staining

Fresh mouse splenocytes were seeded at 2.5 × 10^6^ cells/mL in 12-well plates and stimulated for 24 h with 50 µg/mL OVA or 10 µg/mL p24. Cytokine secretion was blocked by Brefeldin A treatment (GolgiPlug, BD Biosciences) for 17 h; cells were then fixed and permeabilized using GolgiStop kit (BD Biosciences). Cells were stained with CD3-PECy7, CD4-APCCy7, CD8-APC and IFN-γ-PE antibodies (Biolegend) for 1 h at 4 °C in PBS + 1% BSA. Intracellular expression of IFN-γ was determined by flow cytometry.

### Statistical analysis

Data are expressed as mean ± s.e.m. (standard error of the mean). *In vivo* data were compared between groups using a 1-way ANOVA (Analysis Of Variance) test followed by Fisher’s protected least significant difference method (immunogenicity studies in mice) or a 2-way ANOVA test followed by the Holm–Sidak method (immunogenicity studies in NHP) for pairwise multiple comparisons.

## Electronic supplementary material

SUPPLEMENTAL INFORMATION

## Data Availability

Relevant data are available from the corresponding author upon reasonable request.

## Electronic supplementary material

**Supplementary information** accompanies the paper on the *npj Vaccines* website (10.1038/s41541-018-0086-0).
