# Supplementary material for: Overcoming immunogenicity issues of HIV p24 antigen by the use of innovative nanostructured lipid carriers as delivery systems: evidences in mice and non-human primates
Source: NPJ Vaccines. 2018 Oct 1;3:46. doi: 10.1038/s41541-018-0086-0 (PMC6167354; doi:10.1038/s41541-018-0086-0)
Supplement: Supplementary file 1 — SUPPLEMENTAL INFORMATION [file 41541_2018_86_MOESM1_ESM.pdf]

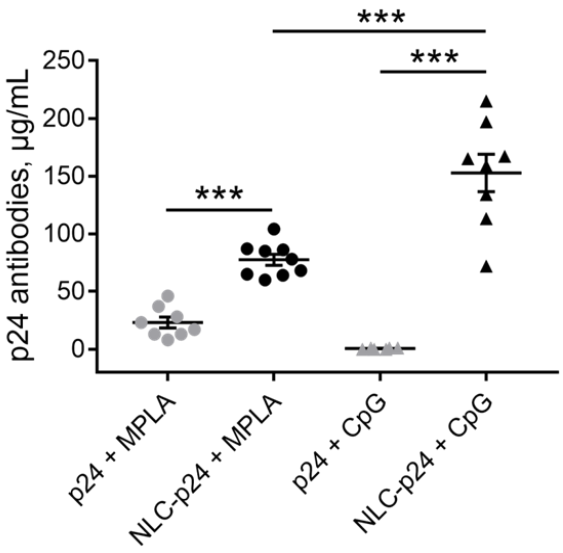

Figure S1. *CpG as immunostimulant results in significantly higher antibody production in mice immunized with p24 formulations compared to MPLA. Humoral response in mice following immunization with 5 µg of p24 in combination with 10 µg of CpG or 10 µg of MPLA, as measured based on total anti-p24 antibody levels in mouse serum. Each point represents an individual mouse, horizontal bars represent the mean for the group and vertical bars indicate the standard error of the mean. Data were compared between groups using a 1-way ANOVA test followed by a Fisher's protected least significant difference test. *** p<0.001.*

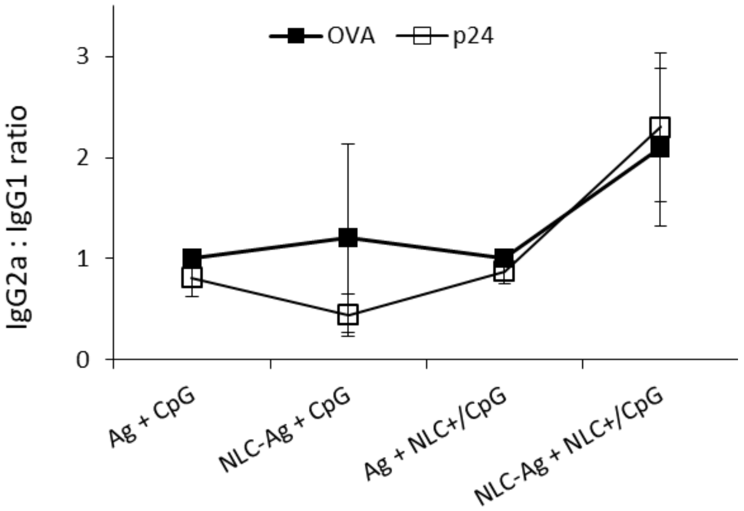

Figure S2. *Antigen-specific IgG2a:IgG1 ratio measured in mice immunized with 10 µg of OVA and 5 µg of p24 antigens with 10 µg of CpG as adjuvant. Squares represent the mean for the group and bars indicate the standard error of the mean.*
